# Socio-Demographic and Clinical Characteristics of Adults With Psychotic Symptomatology Under Involuntary Admission and Readmission for Compulsory Treatment in a Referral Psychiatric Hospital in Cyprus

**DOI:** 10.3389/fpsyt.2021.602274

**Published:** 2021-02-17

**Authors:** Katerina Kaikoushi, Nicos Middleton, Andeas Chatzittofis, Evanthia Bella, Giorgos Alevizopoulos, Maria Karanikola

**Affiliations:** ^1^Cyprus Nursing Services, Ministry of Health, Nicosia, Cyprus; ^2^Nursing Department, Faculty of Health Sciences, Cyprus University of Technology, Limassol, Cyprus; ^3^Medical School, University of Cyprus, Nicosia, Cyprus; ^4^Private Practice, Limassol, Cyprus; ^5^Psychiatric Clinic, Agioi Anargyroi Hospital, Faculty of Nursing, National and Kapodistrian University of Athens, Athens, Greece

**Keywords:** compulsory treatment, involuntary, psychosis, non-adherence, readmission, Cyprus, demographic, coercion

## Abstract

Socio-demographic and clinical characteristics of adults under compulsory psychiatric treatment, have not been reported adequately in Southern European countries. We investigated the socio-demographic and clinical characteristics of adults with psychotic symptomatology who were involuntarily treated in the acute Mental Health Services in Cyprus. A descriptive cross-sectional study was applied. Data collection (December 2016 to February 2018) achieved via a structured questionnaire including demographic and clinical variables. Census sampling was applied in Cyprus referral center for compulsory psychiatric treatment. The sample included 406 individuals (262 males, 144 females). Approximately 86.2% were single, 77.6% were unemployed, and 24.9% held a bachelor's degree. The most frequent clinical diagnosis was schizophrenia or a relevant psychotic disorder (86.4%). The most frequent admission cause was non-adherence to pharmacotherapy along with disorganized behavior (agitation and/or self-care deficit, and/or aggressive behavior, and/or suicidal behavior) (53.6%). Moreover, 70.7% of the sample reported a positive personal history of mental health problems, while 42.1% reported a positive family history of mental health disorders. Half of the participants (52%) were previously involuntarily admitted for compulsory treatment. Adjusted associations of readmission status were reported with Cypriot ethnicity (OR: 4.40, 95%CI: 2.58–7.50), primary education only (OR: 3.70, 95%CI: 1.64–8.37), readmission due to disorganized behavior along with non-adherence to pharmacotherapy (OR: 10.84, 95%CI: 2.69–43.72), as well as along with substance use (OR: 6.39, 95%CI: 1.52–26.82). Readmission was almost five times more likely to occur due to suicidal behavior (OR: 5.01, 95%CI: 1.09–22.99) compared to disorganized behavior not otherwise specified. Additionally, those with a diagnosis of schizophrenia were more than 12 times more frequently readmitted for compulsory treatment compared to other diagnoses (OR 12.15, 95%CI: 1.04–142). Moreover, the participants with higher secondary education had 54.6% less odds to be involuntarily re-admitted compared to Bachelor degree holders (OR 0.442, 95%CI: 0.24–0.79). A high percentage of involuntary treatment was noted due to non-adherence to pharmacotherapy and substance use. Re-evaluation of the effectiveness of relevant community interventions is suggested, as well as implementation of structured educational programs on therapy adherence during psychiatric hospitalization.

## Introduction

Although there is longstanding evidence showing that the incidence of mental disorders in the general population is increasing globally (1), data on the factors associated with severe mental health disturbances leading to compulsory treatment have not been addressed adequately in Southern European and Mediterranean countries (2, 3). Involuntary or compulsory psychiatric treatment is a procedure mainly applied to people with mental disorders lacking their consent, when the intensity of the symptomatology is severe enough to jeopardize personal or social safety (4).

Data on the effectiveness of compulsory treatment are insufficient (5, 6) while self-stigma in those involuntarily hospitalized for compulsory psychiatric treatment has been well-described in international literature (7). At the same time, involuntary hospitalization in mental health settings has been described as a severe stressor for family members of those under compulsory treatment, as well as for healthcare professionals providing care to them (8). Thus, documentation of data which may be applied in policy making, to lessen compulsory hospitalization of people facing mental health problems, may be an important public health issue.

Moreover, a significant proportion of people facing mental health problems, are reluctant to reach formal mental health services for help due to mental health social stigma (9). As a result, their first contact with mental health services is more likely to happen under compulsory hospitalization due to severe deterioration of their mental health status (10). Thus, identification of the clinical and demographic profile of those receiving formal mental health services for the first time, via involuntary hospitalization, may be relevant in formulating awareness interventions in the general population (11). Yet, the socio-demographic and clinical characteristics of individuals under compulsory psychiatric treatment in Southern European countries, including Cyprus, have been understudied (12–14).

Addressing the socio-demographic and clinical characteristics, in those involuntarily admitted in acute mental healthcare settings, may be relevant in developing interventions to empower ill health management in clinical populations and prevent relapse upon their return to everyday life in the community (15). Such data may support interventions regarding treatment options, education and follow up modifications (16, 17). Socio-demographic factors, previously reported in the literature, associated with compulsory treatment are ethnic inequities (3), place of origin, male sex (13), single marital status, receiving welfare reimbursement and unemployment (14), while non-adherence to therapy before hospitalization, poor insight into illness, diagnosis of a bipolar or a schizophrenia-related disorder, positive history of involuntary hospitalizations and risk to others are the clinical factors which have been described in the literature in relation to involuntary hospitalization (14).

### The Context of Cyprus

According to the Psychiatric Compulsory Hospitalization Act of the Republic of Cyprus involuntary hospitalization is deemed as the case of one's admission in a special psychiatric unit for treatment and care following a judicial decree based on the clinical assessment of one psychiatrist without patient's consent [Psychiatric Hospitalization Act of 1997 (77 (I)/1997)] (18). Under this scope, for an involuntary hospitalization to take place all the following three criteria need to be fulfilled: (a) diagnosis of a mental disorder, (b) incapacity of judgment in the interest of one's own health, (c) receiving no treatment either poses a risk for violence against one's self or others, or may severely affect a patient's safety or recovery. According to the present legislation in the Republic of Cyprus, involuntary hospitalization under a judicial decree is provided only in high security units within psychiatric hospitals, i.e., Athalassa Psychiatric Hospital which is the reference center in Cyprus. The duration of involuntary hospitalization ranges from 1 to 28 days, according to the judicial decree and is verified and adjusted accordingly by the assigned psychiatrist at the clinical setting to which the patient is hospitalized (18).

Previous data by the Ministry of Health of Cyprus have shown an upward trend of involuntary admissions in the acute mental health services, per year, since 2007, without, however, mentioning the demographics of this group of service users (19). Furthermore, a literature review in the Pubmed, GoogleScholar, Medline, Embase, and PsycInfo databases did not reveal any epidemiological study in people who are involuntarily admitted for compulsory treatment in the acute mental health services in Cyprus (2, 14), to the best of our knowledge. Additionally, relevant data from Southern European countries and the Mediterranean area are limited (20–22). Therefore, it would be useful to report on the demographics and clinical characteristics of those involuntarily admitted and re-admitted for compulsory treatment in acute mental health services in the cultural context of Cyprus; such data will form a national database on the one hand, and will allow future comparisons with international data, on the other (14). Furthermore, such data may be used in the development of healthcare policy nationally and internationally (23).

## Materials and Methods

### Aim

The aim of the study was to investigate the socio-demographic and clinical characteristics of the adults who were involuntarily hospitalized under compulsory psychiatric treatment in Cyprus. The objectives included testing of the differences regarding clinical characteristics and health-related behaviors (adherence to pharmacotherapy, substance use) in relation to admission and readmission status.

### Research Design

A descriptive correlational study with cross-sectional comparisons was applied.

### Study Environment

The study environment was the Athalassa Psychiatric Hospital (APH). APH is the only inpatient unit of the mental health services of Cyprus where compulsory psychiatric treatment can be provided. The capacity of the APH is 132 beds distributed to one admission unit for males (19 beds), one admission unit for females (19 beds), a high safety nursing ward (2 beds), and three rehabilitation wards (92) covering a population of approximately 875,900 individuals based on the Republic of Cyprus (24). According to the most recent annual report of the Cyprus Ministry of Health (19), 395 individuals are involuntarily hospitalized per year in the APH.

### Sampling Method

A census sampling method was applied for data collection from December 2016 to February 2018. All demographic and clinical data of the adults involuntarily admitted with psychotic symptomatology to the APH during this period were assessed according to the inclusion and exclusion criteria set for this study. The study participants had to be adults who were involuntarily admitted to the APH with psychotic, mood, or substance use-induced symptoms and further diagnosed according to DSM-5 criteria with a psychotic disorder of the spectrum of schizophrenia (e.g., Brief psychotic disorder, Schizoaffective disorder, Delusional disorder, Schizophreniform disorder, etc.), schizophrenia, bipolar, and related disorders, unipolar depressive disorders, substance/medication-induced psychotic and related disorder, substance/medication-induced mood disorder, as well as a dual diagnosis (25). Additionally, the following inclusion criteria were applied: (a) age: 18–65 years; (b) comprehension of the objectives and procedures of the study; (c) signed informed consent for participation in the study; (d) knowledge of the Greek or English language, or available translator to foreigners; (e) longer than 3 days duration of hospitalization.

Individuals with the following characteristics were excluded: (a) neuro-cognitive disorders, such as Alzheimer disease or delirium, (b) intellectual disability, (c) developmental disorders, and (d) personality disorders, based on the clinical diagnosis available during admission. Individuals with personality disorders were not included since the duration of hospitalization of such individuals was 24 h in most of the cases. Yet, individuals with personality disorder characteristics and prominent psychotic or substance use-related symptoms that were hospitalized for more than 72 h were included, since in such cases other diagnostic categories were most frequently applied, e.g., substance/medication-induced psychotic disorder not otherwise specified, or a dual diagnosis.

**Figure 1:**
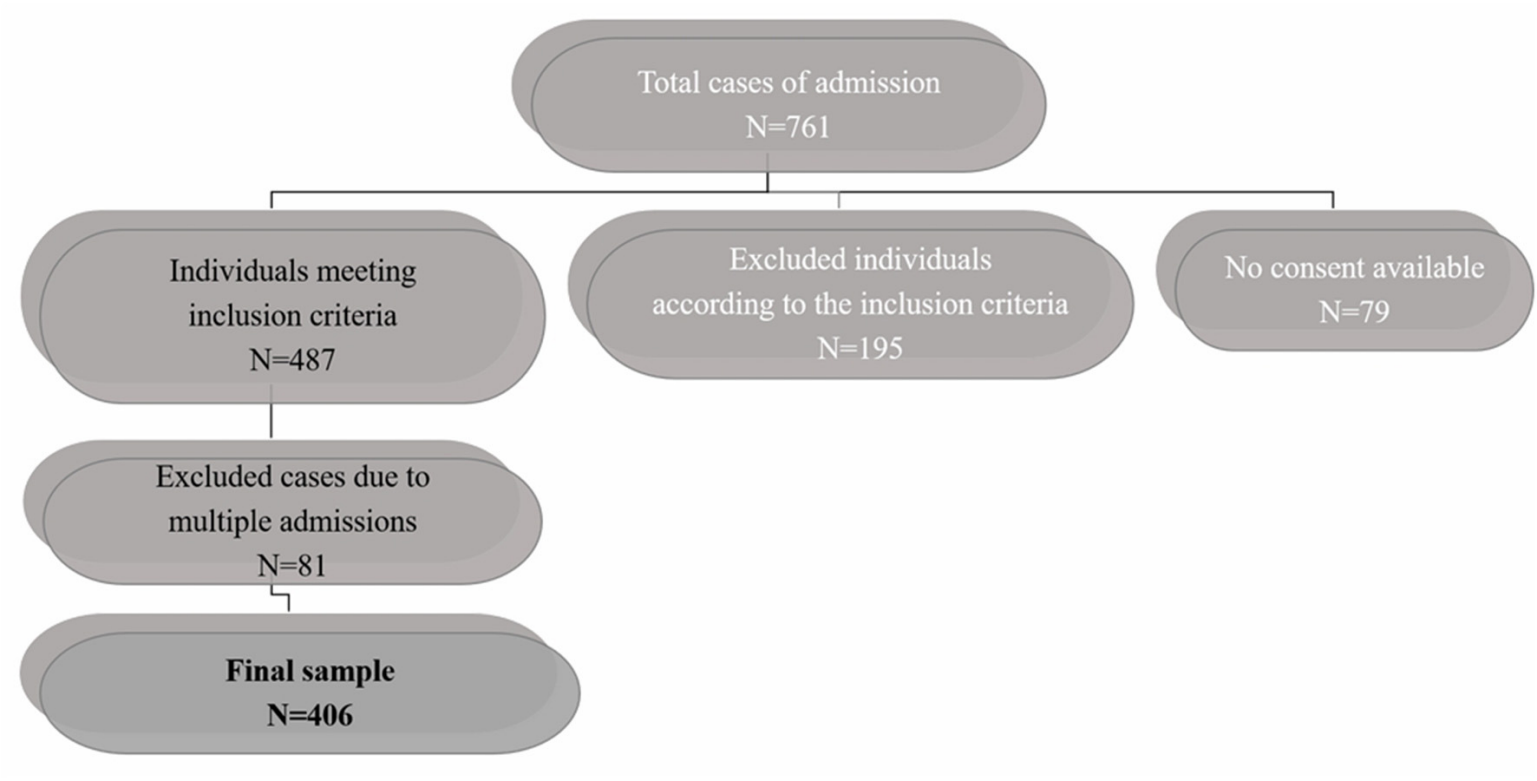
Flow chart of responders.

In total 761 admissions were recorded in the APH. One-hundred and ninety-five cases were rejected because they did not meet the age (*n* = 9 were younger than 18 years and *n* = 13 were older than 65) and diagnosis criteria. The latter were mainly diagnosed with personality disorders (*n* = 152). Twenty-one individuals were diagnosed with intellectual disability. Moreover, 79 people were not included since informed consent was not achieved, either because the level of insight was not adequate at the time of discharge from the APH (*n* = 43), or because the researcher was not present during the discharge, thus she did not have the chance to have a meeting with the patient regarding informed consent process (*n* = 34). Two individuals passed away during their hospitalization. Eighty one over the 487 total cases of individuals meeting the inclusion criteria were repetitively evaluated due to repeated relapses during the study period. These cases were identified according to the question: “Is this your first involuntary admission to APH? (Yes/No).” In those responding with “No,” the records were checked for previous hospitalizations and the sequence of each one was noted. This process was achievable since anonymity was not applied in that phase of the study. As a result, each data collection sheet included information about the number and sequence of previous hospitalizations at the APH, as well as whether a previous assessment by the research team had taken place or not. Therefore, patients with multiple assessments were only included once (first assessment) in the analysis on clinical history and demographic data, comprising. a sample of 406 individuals as depicted in the flowchart (Figure 1).

### Data Collection Process

All individuals admitted in the APH who met the criteria for entry in the study were assessed independently by two members of the research team with advanced clinical experience, i.e., more than 10 years as a specialist (EB, KK). No other researchers were involved in data collection process to minimize relevant bias. The assessment interview took place within the first 72 h after admission and the duration was about 15–20 min. Following, there was a meeting of the two researchers in order to determine the degree of consensus among the two assessments. In case of a disagreement, extensive discussion was followed until a consensus among the researchers was achieved, and a final assessment data sheet was included in the study. Also, the socio-demographic and clinical data were recorded. The data collection sheet, encompassing assessment, socio-demographic, and clinical data, was saved into the file of each responder until signed informed consent was given for inclusion in the present study. The process of obtaining signed informed consent took place on the day of the patient's discharge from the hospital, since by then the responders had achieved adequate level of insight and cognitive functioning to fully understand the information given on the research protocol. The informed consent process lasted for ~25 min. When informed consent was given the data were duplicated and anonymized to be included in the study. Patients who did not provide signed informed consent were not included in the study, yet relevant data remained in their medical file.

The degree of insight was evaluated, via a semi-structured interview. In particular, the degree of patients' awareness on (a) the severity of their illness, (b) the severity of the present relapse symptoms, and (c) the importance of adherence to therapy, were assessed (25). In cases that the degree of insight was assessed as adequate the primary researcher (KK) explained the aim and the objectives of the study, confidentiality issues as well as the fact that the participation was voluntary and irrelevant to the clinical outcome or therapy issues of their ill health.

In the cases where the participants' native language was neither Greek nor English, a 4.3%, a translator was hired to support the process.

A structured questionnaire encompassing socio-demographic and clinical characteristics was used for data collection. Demographic variables incorporated were the following: gender, age, race, nationality, mother language, place of residence, marital status, nationality, educational level, occupational status, and receiving financial reimbursement. The following clinical variables were included: family history of mental illness (psychiatric family history), personal history of mental health problems, current admission diagnosis (psychiatric diagnosis), main relapse symptoms during admission process (main symptomatology). Regarding the present involuntary hospitalization, it was additionally researched to understand if it was considered as the first diagnosed mental health disturbance, or if the participant had experienced additional severe mental health problems prior to the current hospitalization. Additional clinical variable collected were BMI [following measurement of the height (cm) and weight (Kgr) of the participants] and personal history of substance use (type, number, and frequency). Regarding the type of substance use, relevant data were recorded discriminating heavy drugs (cannabis, magic mushrooms cocaine, heroin, ecstasy, etc.) from alcohol use only. Data on substance use considered both past and current use, with an emphasis on the period just before the onset of relapse symptoms. Regarding relapse symptoms, the most prominent, according to the phenomenology of the symptoms and the medical record, was included for data analysis. Regarding current admission diagnosis (psychiatric diagnosis) the following grouping was applied according to the diagnoses reported during the study period: (a) Schizophrenia, (b) Other psychotic disorder of the spectrum of schizophrenia (Brief psychotic disorder, Schizoaffective disorder, Delusional disorder, Schizophreniform disorder, and Substance/medication-induced psychotic disorder), (c) Mood disorders (bipolar and related disorders, unipolar depressive disorder, and Substance/medication-induced mood disorder), (d) Other (Anxiety disorders, Psychotic disorders due to medical condition).

### Ethical Issues of Research

The study protocol was approved by the National Committee of Bioethics of Cyprus (EEBK/EP/2014/08), the Research Committee of the Ministry of Health of the Republic of Cyprus (PN: 5.34:01.7.3E) and the Personal Data Protection Officer (5.43.01.7.6 E, PN: 0237/2014); and conforms to the provisions of the Declaration of Helsinki in 1995 (as revised in Tokyo 2004).

Following comprehensive description of the aim and objectives of the study, the participants provided written informed consent to be included in the study. The voluntary nature of participation, the safety and anonymity of the participants as well as data confidentiality were assured. Also, the participants were informed orally and in writing, through the consent form, of their right to express complaints regarding the objectives and procedures of the study. The researchers involved in the study were registered mental health professionals, and therefore legally obliged to respect data confidentiality and patient safety.

### Data Analysis

Frequencies were tested for categorical variables and the non-parametric test *x*^2^ was applied for comparisons between groups. Moreover, the association between health-related behaviors (admission/re-admission; substance use/no substance use; adherence/non-adherence to pharmacotherapy) and clinical or socio-demographic characteristics under study were investigated using chi-square tests. For all statistical tests, *p*-values of 0.05 or lower were considered statistically significant. Also, odds ratio (and 95% confidence intervals) of health-related behaviors for each of the socio-demographics and clinical characteristics were estimated in logistic regression models. Forward stepwise multivariable logistic regression models were used to select the final set of variables (among a large number) associated with involuntary readmission and psychiatric admission diagnosis (dependent variables, respectively) controlling for the potential confounding effect of the rest of the variables in the final model. For all statistical tests applied in multivariable logistic regression model, *p*-values of 0.05 or lower were considered statistically significant. Data were analyzed through the Statistical Package for Social Sciences (SPSS, Inc., Chicago, IL version 20.00).

## Results

### Demographic Characteristics

The sample included 406 individuals (262 males, 144 females) and the majority belonged to the age group of 45–65 years (33%) and 25–34 years (31.8%). Most of the participants were Greek-Cypriots (72.4%), Greek speaking (82%) and declared “Christian Orthodox” (81.3%). Approximately 86.2% of the participants were single, while the largest percentage of the participants were residents of the Nicosia district (38.4%). Nearly 24.9% of the respondents were holders of a bachelor's degree, while 39.9% had completed higher secondary education. Yet, most of the participants were unemployed (77.6%) and only half of them received state financial reimbursement (49.5%). Table 1 presents a comparison between the demographics of the present sample and data from the general population coming from the most recent census in the Republic of Cyprus (24). According to this comparison, the demographic profile of the responders herein is relevant to the one of individuals from the general population. Yet, some difference was noted. It appeared that single and unemployed males of lower secondary education are more frequently involuntarily hospitalized for compulsory treatment to APH compared to the general population.

**Table 1:** Comparison of socio-demographic characteristics between the sample of the present study (*n* = 406) and the general population of Cyprus.

	**Study sample % (*n*)**	**General population %**
**Gender**		
Male	64.5 (262)	48.6
Female	35.5 (144)	51.4
**Age group**		
20–24	13.1 (53)	7.86
25–34	31.8 (129)	17.11
35–44	22.2 (90)	14.46
45–65	33.0 (134)	24.78
**Ethnicity**		
Greek-Cypriots	72.4 (294)	79.4
Other	27.6 (110)	20.6
**Spoken language**		
Greek	82.0 (333)	80.8
Other	18.0 (73)	19.2
**Religion**		
Christian Orthodox	81.3 (330)	89.0
Other	18.7 (76)	10.9
**Marital Status**		
Married	13.8 (56)	49.9
Single	86.2 (350)	49.23
**Residence**		
Nicosia	38.4 (156)	38.9
Limassol	26.8 (109)	28.0
Larnaca	19.2 (78)	17.0
Paphos	10.1 (41)	10.5
Famagusta	5.4 (22)	5.6
**Educational level**		
Primary school education	14.8 (60)	14.7
Lower secondary school education	20.4 (83)	12.2
Higher secondary school education	39.9 (162)	36.4
Tertiary education	24.9 (101)	30.5
**Vocational status**		
Employed	22.4 (91)	89.0
Unemployed	77.6 (315)	10.9
**Financial reimbursement**		
Yes	49.5(201)	
No	50.5(205)	
**BMI**		
Underweight (<18.5 kg/m^2^)	4.7 (19)	
Normal (18.5–25 kg/m^2^)	40.9 (166)	
Overweight (25–305 kg/m^2^)	26.8 (109)	
Obese (>305 kg/m^2^)	17.2(50)	
**Psychiatric diagnosis**		
Schizophrenia	47.5 (193)	
Other psychotic disorder of the spectrum of schizophrenia	25.4 (103)	
Mood disorder	25.4 (103)	
Other	1.7 (7)	
**Main symptomatology leading to the current involuntary hospitalization**		
Non-adherence to pharmacotherapy and disorganized behavior	53.7 (218)	
Disorganized behavior along with substance use	21.9 (89)	
Suicidal/Self-harming behavior	9.9 (40)	
Aggressive behavior toward others	9.6 (39)	
Disorganized behavior not otherwise specified	4.9 (20)	
**Personal history of involuntary hospitalization**		
First admission	48.0 (195)	
Readmission	52.0 (211)	
**Personal history of mental health problems**		
1st episode of a mental health problem	29.3 (119)	
Positive history of mental health problems	70.7 (287)	
**Psychiatric family history**		
Positive	42.1 (171)	
Negative	43.6 (177)	
Unknown	14.3 (58)	
**Diagnosis of psychiatric family history**		
Schizophrenia and/or other related psychotic disorder	18.2 (74)	
Mood disorder	13.8 (56)	
Schizophrenia/psychotic disorder and substance use	0.5 (2)	
Mood disorder and substance use	1.0 (4)	
Schizophrenia or other psychotic disorder and mood disorder	3.4 (14)	
Other	5.4 (22)	
**Personal substance use history**		
Yes	42.8 (174)	
No	57.2 (232)	
**Dual diagnosis**		
Positive	31.0 (126)	
Negative	69.0 (280)	
**Time of year of the admission**		
Winter	32.0 (130)	
Spring	21.4 (87)	
Summer	29.3 (97)	
Autumn	22.7 (92)	

*The clinical characteristics of the sample are also presented*.

### Clinical Characteristics and Health-Related Behaviors

The most frequent clinical diagnosis in the sample (*n* = 406) was schizophrenia or a psychotic disorder of the spectrum of schizophrenia (86.4%). At the same time, the most frequent admission cause was non-adherence to pharmacotherapy along with disorganized behavior in terms of agitation and/or self-care deficit, and/or aggressive behavior toward others, and/or suicidal behavior (53.6%).

The second most prominent cause of involuntary admission was “disorganized behavior along with substance use” (21.9%). Approximately one out of ten (9.8%) were admitted due to suicidal/self-harming behavior, or because of aggressive behavior against others (9.6%). Approximately 4.9 % of the participants were involuntarily hospitalized because of “disorganized behavior not otherwise specified.”

More than half of the participants, 211 (52%) were previously involuntarily hospitalized for compulsory treatment in the APH, while 195 (48%) participants were admitted at the APH for the first time. Also, ~29.3% of the participants reported that the current episode which led to the involuntary hospitalization was their first diagnosed mental health disturbance. Thus, 70.7% of the sample reported a positive personal history of mental health problems, while 42.1% of the participants reported a positive family history of mental health disorders. The most frequent reported diagnosis of a positive psychiatric family history was schizophrenia or a relevant psychotic disorder (18.2%).

Furthermore, 174 of the participants (42.8%) confirmed a positive personal history of substance use (current or previous use). Among them 46.6% used more than one substance (multi-substances use). Also, almost half of those who reported a positive personal substance use history confirmed daily use of the substance (51.7%), while ~3 out of four fulfilled the criteria for a dual diagnosis (72.4%). Moreover, 83.9% of this subgroup reported use of heavy drugs, i.e., cocaine, heroin, ecstasy, cannabis, etc.

Most of the admissions and re-admissions occurred in the winter (32%) or in summertime (23.9%; Table 1), yet no statistically significant difference was noted in the number of admissions regarding time of year.

### Demographic and Clinical Characteristics of the Participants Who Were Involuntarily Readmitted for Compulsory Treatment at the APH

With regard to the demographic profile the sub-group of the participants who were involuntarily readmitted for compulsory treatment (*n* = 211), the vast majority were Greek-Cypriots (85.8%, *n* = 181), males (66.4%, *n* = 140), single (89.1%, *n* = 188), and 45–64 years old (37%, *n* = 78). They had completed higher secondary education (39.3, *n* = 83); however, they were unemployed (83.9%, *n* = 177) and received financial reimbursement (60.2%). Normal BMI was assessed in the 40.9% of this subgroup.

Among clinical characteristics, the most frequent admission diagnosis was schizophrenia (56.9%, *n* = 120), followed by a psychotic disorder of the spectrum of schizophrenia (19.4%, *n* = 41). The most frequent admission cause was “non-adherence to pharmacotherapy along with disorganized behavior” (66.4%, *n* = 140), while “disorganized behavior along with substance use” was reported in the 20.9% (*n* = 44) of this sub-group. In addition, 68.7% of the entire subgroup had a dual diagnosis. Furthermore, almost one out of five (21.1%) in those readmitted who reported a positive personal history of substance use also reported use of more than one substance.

### Differences Between Admission and Readmission Group

The frequency of readmission compared to the rate of first involuntary hospitalization was higher in white than non-white race participants (96.7 vs. 3.3%, respectively, *p* < 0.001), Cypriots than non-Cypriots (85.8 vs. 14.2%, respectively, *p* < 0.001), as well as Christian Orthodox than non-Christian Orthodox (92.4 vs. 7.6%, respectively, *p* < 0.001) individuals. Similarly, readmission rate was higher in the participants with higher secondary education than in those with tertiary, lower secondary or primary education (39.3 vs. 18.0% and 19.9 and 22.7%, respectively, *p* < 0.001), as well in unemployed participants compared to employed ones (83.9 vs. 16.1%, respectively, *p* = 0.02) and those who received financial reimbursement compared to those who did not (60.2 vs. 39.8%, respectively, *p* < 0.001). Moreover, although those readmitted reported more frequent use of heavy drugs than use of alcohol only (55.1 vs. 44.8%, respectively), this difference was not statistically significant *p* = 0.311), as well as use of multiple substances compared to use of one substance (54.3 vs. 45.7%, respectively, *p* = 0.029). Also, readmitted participants were more frequently involuntarily hospitalized because of non-adherence to pharmacotherapy along with disorganized behavior (66.4%) compared to disorganized behavior along with substance use (20.9%), suicidal/self-harming behavior (8.5%), aggressive behavior toward others (2.8%) or disorganized behavior not otherwise specified (1.4%) (*p* < 0.001). Moreover, readmitted participants had more frequently a diagnosis of schizophrenia (56.9%) compared to other diagnoses, i.e., psychotic disorders of the schizophrenic spectrum (19.4%), mood disorders (23.2%), or other non-specified diagnoses (0.5%) (*p* < 0.001). These data are presented in Table 2.

**Table 2:** Differences in socio-demographic and clinical characteristics between the participants who were for the first time involuntarily admitted for compulsory treatment and those readmitted one or more times (*N* = 406).

**Clinical and socio-demographic characteristics**		**Personal history of involuntary hospitalization**		***x*^**2**^ value**	**df**	***P*-value**
		**1st time admitted participants**	**Readmitted participants**			
Race	White	86.2% (168)	96.7% (204)	14.64	1	0.000
	Other	13.8% (27)	3.3% (7)			
Ethnicity group	Greek-Cypriot	57.9% (113)	85.8% (181)	39.30	1	0.000
	Other	42.1% (82)	14.2% (30)			
Religion	Christian Orthodox	69.2% (135)	92.4% (195)	35.80	1	0.000
	Other	30.8% (60)	7.6% (16)			
Education	Tertiary education	32.3% (63)	18.0% (38)	27.31	3	0.000
	Higher secondary education	40.5% (79)	39.3% (83)			
	Lower secondary education	21.0% (41)	19.9% (42)			
	Primary education	6.2% (12)	22.7% (48)			
Vocational status	Unemployed	70.8% (138)	83.9% (177)	10.02	1	0.002
	Employed	29.2% (57)	16.1% (34)			
Financial reimbursement	Receptors of financial reimbursement	37.9% (74)	60.2% (201)	20.05	1	0.000
	Non receptors of financial reimbursement	62.1% (121)	39.8% (205)			
Number of substances used	More than one substance used	37.8% (31)	54.3% (82)	4.76	1	0.029
	One substance used	62.2% (51)	45.7% (43)			
Main symptomatology leading to the current involuntary hospitalization	Non-adherence to pharmacotherapy and disorganized behavior	40.0% (78)	66.4% (140)	45.97	4	0.000
	Disorganized behavior along with substance use	23.1% (45)	20.9% (44)			
	Suicidal/ self-harming behavior	11.3% (22)	8.5% (18)			
	Aggressive behavior toward others	16.9% (33)	2.8% (6)			
	Disorganized behavior not otherwise specified	8.7% (17)	1.4% (3)			
Psychiatric diagnosis	Schizophrenia	37.4% (73)	56.9% (120)	18.94	3	0.000
	Other psychotic disorder of the spectrum of schizophrenia	31.8% (62)	19.4% (41)			
	Mood disorder	27.7% (54)	23.2% (49)			
	Other	3.1% (6)	1 (0.5)			

These variables were further entered to a multivariable forward stepwise logistic regression analysis and adjusted scores were assessed. In the multivariable analysis, adjusted associations were reported with Cypriot ethnicity (OR: 4.40, 95%CI: 2.58–7.50), primary education only (OR: 3.70, 95%CI: 1.64–8.37), readmission due to disorganized behavior along with non-adherence to pharmacotherapy (OR: 10.84, 95%CI: 2.69–43.72), as well as along with substance use (OR: 6.39, 95%CI: 1.52–26.82). Also, readmission was almost five times more likely to occur due to suicidal behavior (OR: 5.01, 95%CI: 1.09–22.99) compared to disorganized behavior not otherwise specified. Also, those with a diagnosis of schizophrenia were more than 12 times more frequently readmitted for compulsory treatment compared to those with a diagnosis of “other” (OR 12.15, 95%CI: 1.04–142) (see Table 1 for the diagnosis of “other”). Moreover, the participants with higher secondary education had 54.6% less odds to be involuntarily re-admitted compared to Bachelor's degree holders (*B* = −0.815; OR 0.442, 95%CI: 0.24–0.79). These data are presented in Table 3.

**Table 3:** Adjusted odds ratios (and 95%CI) of readmission by clinical and socio-demographic characteristics as estimated in multivariable forward stepwise logistic regression analysis (*n* = 406).

**Clinical and socio-demographic characteristics**	**B**	**S.E**.	**Wald**	**Df**	**Adjusted OR 95%CI**			***p-*values**
**Ethnicity group**								
Non-Cypriot								
**Cypriot**	1.482	0.272	29.627	1	4.403	2.582	7.508	0.000
**Main symptomatology leading to current involuntary admission**								
Disorganized behavior not otherwise specified								
**Non-adherence to pharmacotherapy AND disorganized behavior**	2.383	0.712	11.219	1	10.840	2.688	43.720	0.001
**Education**								
Tertiary								
**Primary**	1.309	0.416	9.894	1	3.702	1.638	8.367	0.002
**Main symptomatology leading to current involuntary admission**								
Disorganized behavior not otherwise specified								
**Disorganized behavior and substance use**	1.855	0.732	6.421	1	6.390	1.522	26.823	0.011
**Main symptomatology leading to current involuntary admission**								
Disorganized behavior not otherwise specified								
**Suicidal behavior**	1.612	0.777	4.301	1	5.012	1.092	22.992	038
**Educational level**								
Tertiary education								
**Higher secondary education**	−0.789	0.294	7.227	1	0.445	0.255	0.807	0.007
**Psychiatric diagnosis**								
Other								
**Schizophrenia**	2.497	1.256	3.956	1	12.151	1.037	142.385	0.047
**Constant**	−5.092	1.450	12.337	1		0.000		0.006

## Discussion

This study presents, for the first time, the socio-demographic and clinical characteristics of those involuntarily hospitalized for compulsory treatment in Cyprus, with special focus on the factors associated with readmission. These data are the first to be recorded at a national level, while international studies on the subject coming from the Southern European and Mediterranean countries are limited (23, 26). Comprehension of the factors related to acute symptomatology of severe mental disorders is a major public health issue and of paramount importance for clinical practice (3, 14).

Regarding the socio-demographic profile of those involuntarily admitted for compulsory treatment in Cyprus the present study reports that single and unemployed males of secondary education are more frequently involuntarily admitted for compulsory psychiatric treatment compared to the general population (24).

Nevertheless, the most important finding herein regards readmission rate, and related factors. Specifically, slightly more than half of the participants reported a positive history of involuntary hospitalizations for compulsory treatment. Other researchers report that ~20–40% of mental health service users are repeatedly involuntarily hospitalized for compulsory treatment (4, 27, 28). Frequent, repetitive involuntary hospitalizations seem to be an important clinical problem in Cyprus, as well as internationally, since it seems that a large proportion of mental health service users fail to remain in remission for a long time. This underlines the need for more effective relapse preventive interventions, mainly in community level (29).

The present study revealed that the most frequent cause for involuntary hospitalization, regarding either first admission or readmission, was non-adherence to pharmacotherapy along with disorganized behavior. Moreover, this factor seemed to be the most important clinical risk factor for readmission. Specifically, those with disorganized behavior linked with non-adherence to pharmacotherapy were almost 11 times more likely to be involuntarily readmitted compared to those with disorganized behavior not related to any specific cause. This is in line with international literature, since the main cause of relapse among people with severe mental health problems internationally seems to be non-adherence to pharmacotherapy (2–4, 27, 28). Thus, emphasis needs to be given to educational programs in pharmacotherapy and treatment options in order to enhance adherence to therapy (30–34). Moreover, the present study presents additional risk factors for readmission for compulsory treatment in Cyprus. Thus, according to the present results preventive measures on readmission need to focus on Greek-Cypriots of primary education, with a positive personal history of substance use and non-adherence to pharmacotherapy, and a diagnosis of schizophrenia. Specifically, it was shown that those who were admitted due to disorganized behavior along with substance use were more than six times more frequently readmitted compared to those admitted due to disorganized behavior not otherwise specified. Also, the participants with primary education only, were almost four times more frequently involuntarily readmitted compared to Bachelor's degree holders, as did Greek-Cypriot who were more than four time more likely to be readmitted compared to foreigners; also, those with a diagnosis of schizophrenia were more than 12 times more frequently readmitted compared to those with other diagnoses. Moreover, special focus needs to be given on the finding that those with suicidal behavior were five times more likely to be readmitted compared to those with disorganized behavior not otherwise specified. Prevention of suicidality within community settings needs to be a priority of the healthcare system of Cyprus.

Although we did not apply a comparison to those involuntarily readmitted with individuals who voluntarily used acute mental health services, the present data on the profile of those involuntarily admitted for compulsory treatment in Cyprus are in accordance with international literature (14). Yet, further comparison, between the present data and findings from other studies, needs to be completed, considering this limitation. Nevertheless, there are limited data available coming from populations who voluntarily use mental health services or from the general population of Cyprus. For instance, there is only one study available providing data on substance use in the general population of Cyprus (35). In particular, this study conducted in a sample of 1,500 university students coming from all four district areas of Cyprus with an age range between 18 and 40 years, revealed that 4.2% of the participants had a positive history of substance use, a percentage much lower than the one reported herein. Moreover, the most frequently used substance among these students was cannabis. Similarly, the responders herein more frequently reported use of cannabis, while ~1 out of 2 reported a positive history of substance use. Also, among those who reported a positive history of substance use, ~1 out of 2 was a daily user and the vast majority fulfilled criteria for dual diagnosis.

Regarding voluntary psychiatric treatment, there is also only one available study conducted in Cyprus. Specifically, the study by Mitsis (36), held in 100 individuals from the general population who voluntarily received treatment in a private clinic for substance use-related problems, showed that cannabis was one of the most common substances used, while the majority of the participants had a dual diagnosis.

Nevertheless, international data show that substance use is an important clinical factor related to occurrence, as well as recurrence of mental disturbance symptoms (37, 38). In the present study almost one out of two participants either reported substance use directly before escalation of the symptoms, which led to involuntary admission, or reported a positive history of substance use. Overall, although there are several studies that have investigated the relationship between substance use and manifestation of mental health symptoms (37, 38), further investigation into the relationship between substance use and involuntary hospitalization for compulsory treatment is suggested, considering the multifactorial relationship between these two phenomena and their inherent difficulties.

An additional important finding reported herein is that one out of three of the participants reported that the current hospitalization was the first diagnosed severe mental health episode. This data implies that individuals, even when faced with disturbing mental health symptoms, are either not aware of their severity and need for treatment or reluctant to voluntarily seek for help. A possible explanation may be relevant to mental health social stigma. This has been shown to constitute a severe barrier against help seeking behaviors for mental health problems in Cyprus (7). Moreover, mental health illiteracy and limited awareness on early symptoms of mental health problems, or limited access to mental health services may be also involved (39). Nevertheless, this finding is alarming regarding healthcare preventive policy in Cyprus, while at the same time may support suggestions for further research on the subject.

With regard to the demographics, male gender has been well-documented in international literature regarding the demographic-related factors associated with involuntary admission and subsequent compulsory treatment (3, 14, 26, 27, 34, 40–43). Yet, these data come from studies comparing populations under involuntary therapy with others under voluntary treatment. In the present study such a comparison was not applied, however, male gender was more frequent in the sample, and this frequency was higher compared to the general population. At the same time, there are studies which report increased frequency of compulsory treatment among females (13, 27, 28, 44–46). These data mainly come from non-European countries, i.e., USA, Croatia, China, Holland, and Brazil. Regarding the APH, the referral hospital of Cyprus for compulsory treatment, although there is equal number of beds for males and females at the acute units for compulsory treatment, most of the times the capacity in male units is full while there are non-occupied beds in female units (anecdotal data).

The present study also showed that the majority of those involuntarily admitted at the APH were singles. Indeed, international literature poses singles with no supportive family or social network in increased risk for compulsory treatment (2, 3, 14, 47, 48). However, one study in Bangladesh showed that the majority of those under compulsory treatment (53.1%) were married (49).

Furthermore, most of the participants herein had completed higher secondary education. Also, one out of four participants had completed tertiary education. These are in accordance with data from the general population of Cyprus, and they may also suggest that despite severe mental illness the participants were able to proceed with their studies. On this basis, it could be considered that, compared to the past or other countries, the diagnosis of severe mental disorder in Cyprus takes place at an early stage, and subsequent effective treatment is applied, thus restraining possible cognitive implications resulting from a severe mental disorder (25, 50). Nevertheless, there is contradictory data in studies conducted in South Asia (Bangladesh), Italy, Turkey, China, Switzerland, Norway, and Greece according to which the largest percentage of those involuntarily admitted for compulsory treatment have only primary education (20, 40, 41, 45, 51).

The increased frequency of unemployed in the present study is supported by international data (14), which highlight unemployment as a risk factor for involuntary hospitalization. These findings reveal the importance of integrating professional specialization programs in rehabilitation services, enabling the participants to acquire vocational skills and further prevent occupational exclusion, social isolation and self-stigmatization (52, 53). In the present study although most of the participants had completed higher secondary education, the majority of them were unemployed. Therefore, more extensive and inclusive vocational specialization/post-graduate education programs are proposed, in terms of the number of trainees and type of specialization provided to individuals with severe mental health problems. Participation in these programs should commence during hospitalization and be further enhanced after discharge and during life in the community with focus on retaining a job. Overall, education in professional skills and the necessity for effective vocational rehabilitation programs needs to become a priority for healthcare systems, since there are data which show that unemployment is a severe stressor in people with mental health problems. Indeed, unemployed with financial problems seem to be more frequently involuntarily admitted for compulsory treatment (3, 13, 22, 28, 54, 55). Only two studies, one in Brazil and one in China, showed that the majority of those involuntarily hospitalized for compulsory treatment were employed (44, 45). Thus, further investigation into culture specific, gender-related factors associated with these data is considered necessary.

### Proposed Interventions

Development of clinical guidelines regarding preventive interventions in community settings for substance use and non-adherence to therapy in clinical populations are proposed. Also, educational programs on adherence to therapy regarding pharmacotherapy and psycho-social interventions need to be implemented during hospitalization, as well as in community settings. Core elements of these programs should be empowerment for participation in clinical decision-making regarding treatment options, as well as capacity building on self-management ill health skills, including effective management of pharmacotherapy. Relevant programs are scarce in Cyprus, mainly administered in the private sector.

Additionally, reform of the present legislation on compulsory hospitalization in Cyprus may be needed, aiming to restrict the causes of involuntary hospitalization. Furthermore, one may suggest integration of mandatory monitoring in community settings by mental health professionals. This reform may be suitable for those individuals who although are in relapse or in acute phase, are not directly harmful to themselves or others, while due to decreased or lack of insight refuse to follow a therapeutic plan. Relevant legislation reforms regarding compulsory treatment have been established in Portugal and Australia (56–58). Yet, further studies on the effectiveness of these programs are needed (59). Most importantly, interventions to minimize the stigma and increase access to formal mental health care are proposed.

Acute short-term residential treatment programs for dual diagnosis are also proposed to be implemented in Cyprus, aiming to provide simultaneous treatment for mental disturbance symptoms and substance-use related problems (2). Also, during the implementation of compulsory substance use treatment it is necessary for the clinicians and healthcare policy makers to consider the cultural context of Cyprus and the data reported herein regarding target populations and duration of programs.

### Limitations

Data collection took place in a 14 months period which may have jeopardized the generalizability of the present findings. However, since approximately 17% of the participants were readmitted in the APH this may suggest that a precise representation of those involuntarily admitted for compulsory treatment has been achieved. In other studies, the time period for data collection was mainly 1–8 years (2, 3, 14). Moreover, in the case when an individual was admitted multiple times, only the first assessment was included in order to avoid duplication of demographic data. Yet, since data were anonymized, it was not possible to identify those who were admitted multiple times, therefore assess differences in the symptomatology and severity of symptoms in multiple relapses and include relevant data in the present study.

Most importantly, the cross-sectional design of the present study does not allow any inference in relation to the direction of the observed associations. At least with regards to factors such as positive history of substance use or family history of mental disorders causality may be assumed. Overall, cross-national comparisons are difficult due to diverse healthcare systems and legislation norms regarding compulsory treatment for mental health problems, however there is a need for collaborative international studies to explore the prevalence of involuntary hospitalization and involuntary readmissions across different settings and cultures employing common assessment tools and standard methodology. Nevertheless, the large sample in this study and the use of a structured clinical assessment procedure triangulated by two independent researchers permits an accurate estimation of the data in this population.

## Conclusions

The results of the present study confirm, to a certain degree, previous international and limited local data regarding clinical and socio-demographic factors associated with involuntary hospitalization for compulsory treatment in people with severe mental health problems. By recording the clinical and socio-demographic characteristics of those under compulsory psychiatric treatment, a deeper comprehension of their profile is achieved within the cultural context of Cyprus. Further studies focusing on the clinical and socio-demographic characteristics of severely mentally ill individuals are proposed in order to develop and implement Clinical Guidelines focused on primary, secondary, and tertiary prevention issues. Further studies associating biological markers with clinical and socio-demographic characteristics in clinical populations under compulsory treatment for severe mental health problems are also proposed.

## Data Availability Statement

The raw data supporting the conclusions of this article will be made available by the authors, without undue reservation.

## Ethics Statement

This study was reviewed and approved by the National Committee of Bioethics of Cyprus (EEBK/EP/2014/08), Research Committee of the Ministry of Health of the Republic of Cyprus (PN: 5.34:01.7.3E), and the Personal Data Protection Officer (5.43.01.7.6 E, PN: 0237/2014). The patients/participants provided their written informed consent to participate in this study.

## Author Contributions

This study is part of the Ph.D. work of KK, who participated in study design, data collection, data analysis, and drafted the manuscript. NM participated in study design, data analysis, and interpretation of the results. AC participated in data interpretation. EB participated in data collection and data interpretation. GA participated in study design. MK participated in study design, data analysis, interpretation of data, writing the manuscript and also she was the supervisor of KK, from which this manuscript was produced. All authors have read and approved the final manuscript.

## Conflict of Interest

The authors declare that the research was conducted in the absence of any commercial or financial relationships that could be construed as a potential conflict of interest.
